# Supplementary material for: A yeast phenomic model for the influence of Warburg metabolism on genetic buffering of doxorubicin
Source: Cancer Metab. 2019 Oct 23;7:9. doi: 10.1186/s40170-019-0201-3 (PMC6806529; doi:10.1186/s40170-019-0201-3)
Supplement: Supplementary file 9 — Additional file 9. GO term-specific heatmaps for REMc/GTF-enriched clusters. GO term-specific heatmaps for significant GO process terms were generated as described in methods and Figs. 3 and 4. Any related child terms are presented in subsequent pages of the parent file name. GO terms with more than 100 children, with 2 or fewer genes annotated to the term, or a file size over 300KB are not shown. All heatmaps are generated with the same layout (see Figs. 3 and 4). [file 40170_2019_201_MOESM9_ESM.bz2 › Additional_File9_GOTermHeatmaps/Additional_File9_GOTermHeatmap/ribosomal_large_subunit_biogenesis.pdf]

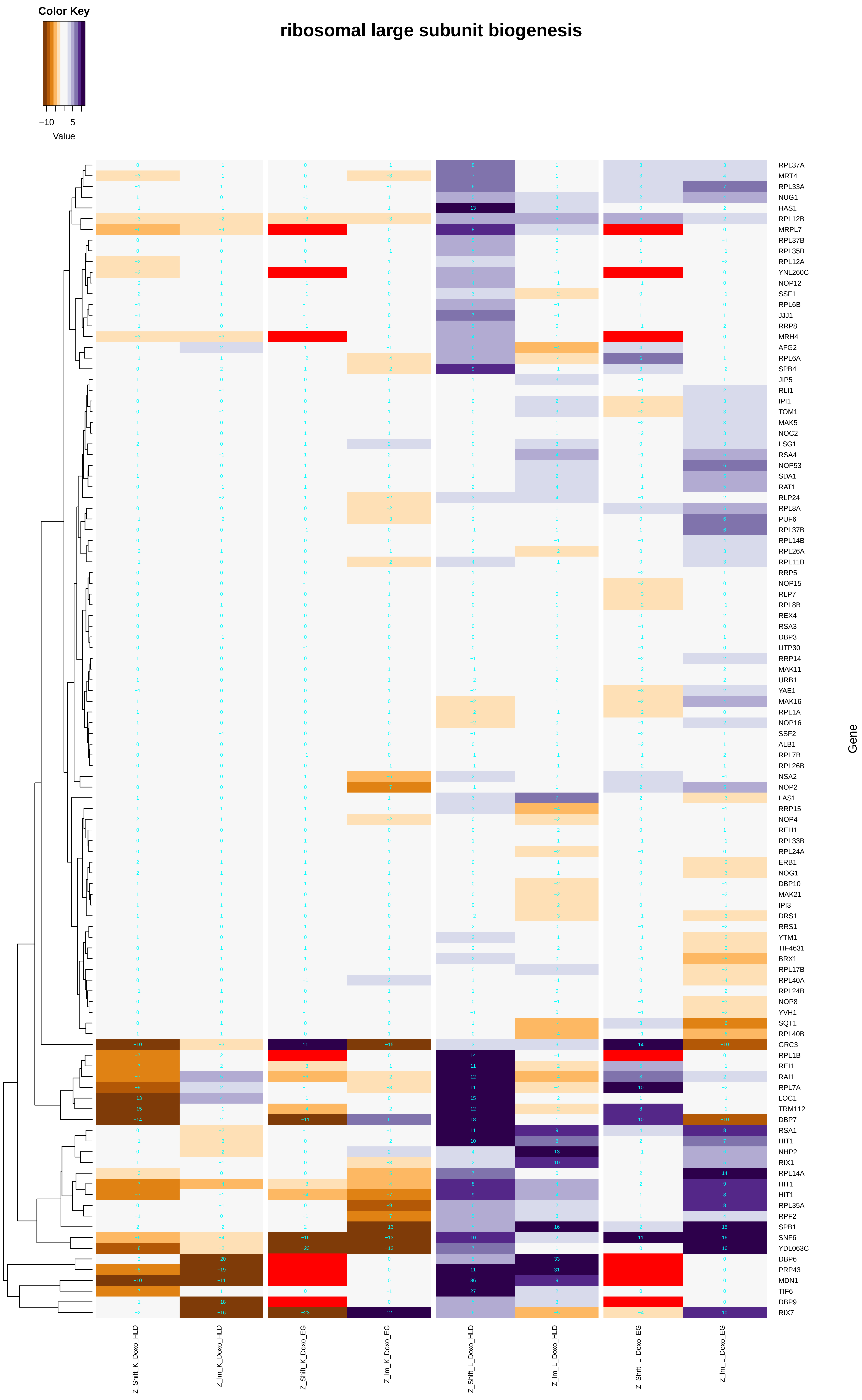

ribosomal large subunit biogenesis
Color Key
−10
5
Value
Gene
RPL37A
MRT4
RPL33A
NUG1
HAS1
RPL12B
MRPL7
RPL37B
RPL35B
RPL12A
YNL260C
NOP12
SSF1
RPL6B
JJJ1
RRP8
MRH4
AFG2
RPL6A
SPB4
JIP5
RLI1
IPI1
TOM1
MAK5
NOC2
LSG1
RSA4
NOP53
SDA1
RAT1
RLP24
RPL8A
PUF6
RPL37B
RPL14B
RPL26A
RPL11B
RRP5
NOP15
RLP7
RPL8B
REX4
RSA3
DBP3
UTP30
RRP14
MAK11
URB1
YAE1
MAK16
RPL1A
NOP16
SSF2
ALB1
RPL7B
RPL26B
NSA2
NOP2
LAS1
RRP15
NOP4
REH1
RPL33B
RPL24A
ERB1
NOG1
DBP10
MAK21
IPI3
DRS1
RRS1
YTM1
TIF4631
BRX1
RPL17B
RPL40A
RPL24B
NOP8
YVH1
SQT1
RPL40B
GRC3
RPL1B
REI1
RAI1
RPL7A
LOC1
TRM112
DBP7
RSA1
HIT1
NHP2
RIX1
RPL14A
HIT1
HIT1
RPL35A
RPF2
SPB1
SNF6
YDL063C
DBP6
PRP43
MDN1
TIF6
DBP9
RIX7
Z_Shift_K_Doxo_HLD
Z_Im_K_Doxo_HLD
Z_Shift_K_Doxo_EG
Z_Im_K_Doxo_EG
Z_Shift_L_Doxo_HLD
Z_Im_L_Doxo_HLD
Z_Shift_L_Doxo_EG
Z_Im_L_Doxo_EG
